# Unlocking the bacterial condensome: from “dark matter” to design

**DOI:** 10.1091/mbc.E25-08-0397

**Published:** 2026-09-14

**Authors:** Daniel S. Trettel, Claudia A. Mak, Y Hoang

**Affiliations:** ^a^Los Alamos National Laboratory, Bioscience Division, Microbial and Biome Sciences group, Los Alamos, NM, 87545; ^b^Department of Biochemistry and Molecular Biology, SUNY Upstate Medical University, Syracuse, NY, 13210; Harvard University

## Abstract

Biomolecular condensates have transformed our understanding of eukaryotic cellular organization, yet their roles in bacteria remain comparatively underexplored. This gap arises from the physical constraints of bacterial cell size, the diversity of molecular mechanisms that drive condensation, and the limited representation of bacterial condensates in current predictive frameworks. Here, we argue that condensation is a widespread but underrecognized principle of bacterial intracellular organization. We describe how scaffold architecture, multivalent interactions, and non-equilibrium regulation collectively determine condensate material states and cellular function. Using bacterial microcompartments as a case study, we show how diverse condensation mechanisms can converge to organize functional organelles. We further highlight emerging computational, imaging, and perturbation-based approaches that are shifting the field from condensate identification toward mechanistic understanding and predictive design. By uncovering the physical principles governing bacterial condensates, we envision their use as programmable biomaterials whose properties can be engineered to control cellular function and enable synthetic organelles.

## Abstract (executive summary)

- Although condensates represent a widespread principle of intracellular organization in bacteria, they remain underrecognized, with their molecular mechanisms and physiological roles poorly understood due to challenges in prediction, imaging, and mechanistic dissection.
- The bacterial condensome emerges from a continuum of physical mechanisms, where scaffold architecture, multivalent interactions, and non-equilibrium regulation collectively shape cellular organization and function.
- Advances in computation, imaging, and synthetic biology are transforming bacterial condensates from elusive biological phenomena into programmable biomaterials for engineering synthetic organelles and controlling cellular functions.

## INTRODUCTION

Life depends on the ability of cells to organize their crowded cytoplasm. While membrane-bound compartments achieve separation through physical diffusion barriers, bacteria are increasingly found to utilize biomolecular condensates (hereafter condensates) for spatiotemporal control (Azaldegui *et al.*, 2021). Condensates arise when multivalent interactions concentrate specific biomolecules into dynamic mesoscale assemblies that regulate physiology (Banani *et al.*, 2017; Cohan and Pappu, 2020). Much like cosmological dark matter, which constitutes a major component of the universe yet is inferred primarily through its effects on galaxies, biomolecular condensates may represent a widespread but underrecognized principle of intracellular organization in bacteria, with their prevalence and biological functions often inferred from converging biophysical and cellular evidence rather than direct visualization alone.

In this Perspective, we outline the molecular grammar underlying bacterial condensation and argue that these mechanisms are best viewed as a continuum rather than discrete categories. Using bacterial microcompartments as a case study, we illustrate how these mechanisms together organize bacterial physiology. We then discuss the evidence standards, challenges, and tools required to illuminate this “bacterial dark matter.” Finally, we address how mechanistic insight is beginning to enable predictive condensate engineering.

## THE MOLECULAR GRAMMAR OF THE BACTERIAL CONDENSOME

The molecular logic of condensates frequently follows a “stickers and spacers” framework. Here, scaffolds are biomolecules that provide the multivalent interaction network driving condensate assembly. Within these scaffolds, interaction motifs (stickers) provide valency for reversible crosslinks, while flexible regions (spacers) modulate solubility and material state (Figure 1A; Borcherds *et al.*, 2021; Ginell and Holehouse, 2023). Scaffold properties, like sticker and spacer sequence and patterning (Das and Pappu, 2013; Quiroz and Chilkoti, 2015; Wang *et al.*, 2018; Dzuricky *et al.*, 2020; Martin *et al.*, 2020; Dai *et al.*, 2023a), establish the biophysical landscape for condensation by defining the concentrations of saturation (C_sat_) and percolation (C_perc_). Scaffolds can also selectively recruit low valency “clients” which influence phase behavior (Banani *et al.*, 2016; Espinosa *et al.*, 2020; Rana *et al.*, 2024; Alberti *et al.*, 2025). Together, these multivalent interactions can generate a spectrum of organizational regimes: density-driven liquid-liquid phase separation (LLPS), connectivity-driven percolation, which drives gelation without demixing, and phase separation coupled to percolation (PSCP; Mittag and Pappu, 2022), in which the interplay between density and network formation determines material state (Table 1; Figure 1B). Condensate location, size, and concentration threshold can be further regulated by energy flux (i.e., NTP hydrolysis and chemical reactions; Wurtz and Lee, 2018; Hondele *et al.*, 2019; Hondele *et al.*, 2020) and nucleation surfaces (i.e., membranes or nucleoid bridging; Broedersz *et al.*, 2014; Hernández-Vega *et al.*, 2017; Heinkel *et al.*, 2019; Lasker *et al.*, 2020; Ryu *et al.*, 2021; Morin *et al.*, 2022; Snead *et al.*, 2022; Hsu *et al.*, 2023; Park *et al.*, 2024; Singh *et al.*, 2025). These observations reinforce our central argument that bacterial condensates should be understood as a continuum of physical mechanisms rather than discrete assembly classes.

**FIGURE 1:**
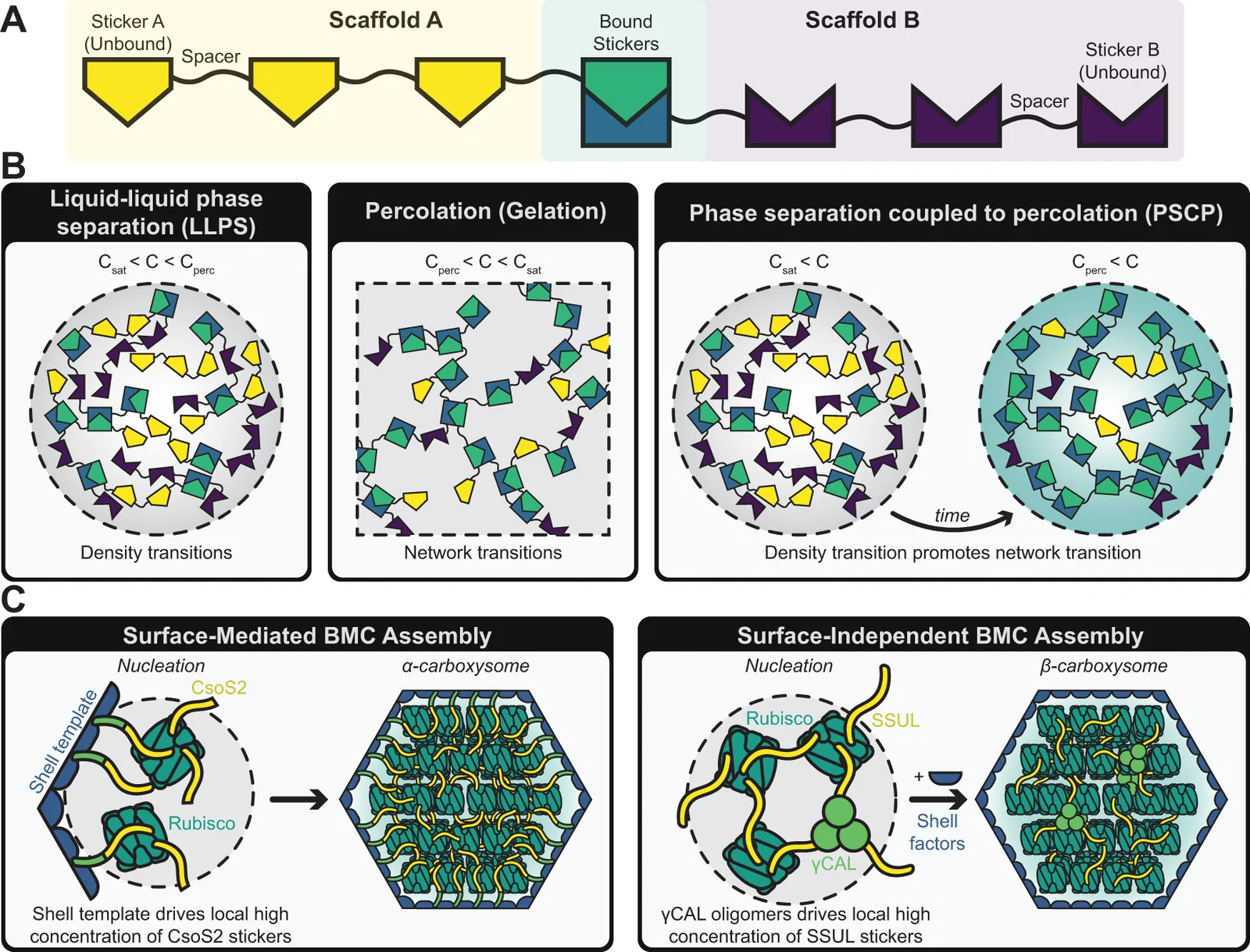
The molecular grammar and biophysical regimes of bacterial condensates. (A) Condensate assembly is driven by multivalent scaffolds featuring associative interaction motifs (stickers) and flexible regions (spacers). (B) Condensate topology is governed by the interplay between saturation (C_sat_) and percolation (C_perc_) concentrations. Density-driven transitions yield liquid-liquid phase separation (LLPS), while connectivity-driven network transitions result in system-spanning percolation. In phase separation coupled to percolation (PSCP), an initial density transition creates a local environment exceeding C_perc_, driving time-dependent network maturation. (C) Carboxysomes demonstrate contrasting nucleation strategies to concentrate multivalent interactions. (Left) α-carboxysome assembly is surface-mediated, where a pre-formed shell template concentrates the multivalent CsoS2 scaffold to link Rubisco to the interior. Note that CsoS2 is translated in a short (CsoS2A) and long (CsoS2B) form with multivalent shell-binding repeats (green). (Right) β-carboxysome assembly is surface-independent, driven by γCAL oligomers that concentrate multivalent SSUL domains to condense the core before shell encapsulation.

**TABLE 1:** The distinct phase separation mechanisms produced by multivalent biomolecules.

Mechanism	Primary driver	Parameters	Molecular mobility^*^	Material result	Bacterial examples
Liquid-liquid phase separation (LLPS)	Thermodynamic demixing (density transitions) (Harmon *et al.*, 2017; Mittag and Pappu, 2022)	C > C_sat_ (Mittag and Pappu, 2022)	High	Simple fluid (Newtonian)	BR-bodies (Al-Husini *et al.*, 2018), RNAP clusters (Weng *et al.*, 2019; Ladouceur *et al.*, 2020), ParB-*parS* (Broedersz *et al.*, 2014; Guilhas *et al.*, 2020)
Percolation (gelation)	Network connectivity (network transitions) (Mittag and Pappu, 2022)	C > C_perc_ (Mittag and Pappu, 2022)	Low	Physical gel	Cytoplasm at large (Parry *et al.*, 2014)
Phase separation coupled to percolation (PSCP)	Network connectivity coupled to thermodynamic demixing (Harmon *et al.*, 2017; Mittag and Pappu, 2022)	C > C_sat_, the local C > C_perc_ (Harmon *et al.*, 2017)	Restricted	Viscoelastic fluid (non-Newtonian) (Alshareedah *et al.*, 2021; Mittag and Pappu, 2022)	BMC (Wang *et al.*, 2019; Oltrogge *et al.*, 2020; Trettel *et al.*, 2025b), PopZ (Lasker *et al.*, 2022), McdB (Basalla *et al.*, 2023), CsgF/CsgB (Swasthi *et al.*, 2023), aggresomes (Pei *et al.*, 2025), FtsZ-SlmA (Monterroso *et al.*, 2019)

^*^Molecular mobility rankings are relative, dependent on experimental conditions and composition, with no universal consensus for thresholds.

Bacterial microcompartments (BMCs) illustrate how multiple condensation mechanisms can be integrated within a single organelle. BMCs, including α- and β-carboxysomes, are < 300 nm polyhedral protein shells that encapsulate concentrated enzymatic cores (Kerfeld *et al.*, 2018). Their biogenesis proceeds through condensate-like intermediates formed via multivalent scaffold-cargo interactions (Figure 1C; Cameron *et al.*, 2013; Chen *et al.*, 2013; Wang *et al.*, 2019; Oltrogge *et al.*, 2020; Zang *et al.*, 2021; Yang *et al.*, 2022; Trettel *et al.*, 2025b; Yang *et al.*, 2025; Zang *et al.*, 2026). The distinct molecular grammars of the scaffolds prevent hybrid core formation (Chang *et al.*, 2026), while scaffold modifications can alter BMC morphology, dynamics, and assembly mode (whether shell- or cargo-nucleated; Mohajerani *et al.*, 2021; Tsidilkovski *et al.*, 2022; Yang *et al.*, 2022; Oltrogge *et al.*, 2023; Trettel *et al.*, 2025b). These observations demonstrate how changes in molecular architecture propagate to higher-order cellular organization.

Despite their distinct assembly mechanisms, BMCs demonstrate material-state changes characteristic of glassy dynamics and PSCP. For instance, the high concentration of compartmentalized cargo promotes low-affinity cargo–cargo binding events that increase with concentration and create glassy ordering within particles (Metskas *et al.*, 2022; Ni *et al.*, 2022; Trettel *et al.*, 2022; Evans *et al.*, 2023; Kong *et al.*, 2024). These material changes promote high packing densities (Sun *et al.*, 2022; Sun *et al.*, 2024) without sacrificing substrate access (Metskas *et al.*, 2022). While mechanistically distinct, PSCP has also been recognized in some BMC intermediates (Trettel *et al.*, 2025b). Further, the internal dynamics of scaffold-cargo condensates demonstrate environmental responsiveness, such as redox-dependence, likely by promoting network rearrangements (Turnšek *et al.*, 2024; Zang *et al.*, 2026). The functional consequences of material transitions are mirrored in other bacterial condensates, like aggresome compaction regulating mRNA protection, suggesting that material-state regulation is a general organizational strategy (Pei *et al.*, 2025).

BMCs also exemplify how condensation can be regulated by non-equilibrium processes. In the carboxysome McdAB positioning system, McdB, a condensate-forming protein, connects carboxysomes to McdA, a nucleoid-bound ATPase (MacCready and Vecchiarelli, 2021). McdB bound to carboxysomes stimulates McdA ATPase activity, triggering its dissociation from the nucleoid and establishing a dissipative steady-state that prevents carboxysome clustering and contributes to BMC morphology, composition, and function (Hakim *et al.*, 2021; Rillema *et al.*, 2021; Hoang *et al.*, 2025). Dissipative steady-states are leveraged by other bacterial positioning systems, like ParABS for plasmid inheritance (Graham *et al.*, 2014; Vecchiarelli *et al.*, 2014; Fisher *et al.*, 2017; Babl *et al.*, 2022). More broadly, this illustrates how condensation mechanisms can be integrated with active cellular processes to produce behaviors that cannot be explained by equilibrium assembly alone.

The role of the BMC shell remains debated. Because many BMC enzymes remain functional without a shell (Fan *et al.*, 2010; Aussignargues *et al.*, 2015; Mills *et al.*, 2022; Yang *et al.*, 2022), and shells display environmental responsiveness (Sutter *et al.*, 2016; Fuentes-Cabrera *et al.*, 2022) as well as limited substrate selectivity (Trettel *et al.*, 2022; Sarkar *et al.*, 2024; Yadav *et al.*, 2026), their function likely extends beyond simple containment and protection. We propose that the BMC shell can function as a phase modulator, providing, in some cases, a nucleation surface (Mohajerani *et al.*, 2021) that can also constrain condensate intermediates (Douliez *et al.*, 2019; Gao and Mann, 2023; Lin *et al.*, 2023). By further limiting coalescence (Folkmann *et al.*, 2021; Abeysinghe *et al.*, 2024; Turnšek *et al.*, 2024; Costantino *et al.*, 2025), ripening (Chang *et al.*, 2026), and material aging of the enzymatic core, BMC shells help to maintain optimal size and function. Here, BMCs are not rigid protein containers (Rotskoff and Geissler, 2018) but dynamic organelles (Trettel *et al.*, 2024) whose function emerges from the interplay between scaffold architecture, material state, and non-equilibrium regulation.

In sum, BMCs illustrate a central theme of the bacterial condensome: higher-order cellular organization emerges from multiple distinct yet interdependent physical mechanisms. Distinguishing among these mechanisms remains a major challenge and motivates the development of new experimental and computational approaches. In bacteria, this challenge is amplified by the physical constraints of the cellular environment itself.

## UNDERSTUDIED, BUT NOT UNCOMMON

Studying condensation in bacteria presents unique challenges that largely stem from their small cellular dimensions (∼1 µm compared with ∼10–100 µm for many eukaryotic cells; Hodgins *et al.*, 2025). The reduced cellular volume limits the number of molecules available to participate in assembly and can impose strong finite-size constraints on nucleation (Wedekind *et al.*, 2006). These finite-size effects necessitate higher supersaturation to support initial nucleation. However, this requirement can be compensated by stochastic fluctuations, which are more effective in smaller volumes (Hill, 1962), surface-assisted nucleation that lowers energetic barriers, and the extreme macromolecular crowding of the cytoplasm (∼ six-fold more concentrated than in human cells; Leeb *et al.*, 2020). These biophysical constraints render many bacterial condensates effectively invisible to conventional imaging approaches, reaffirming their characterization as a form of “bacterial dark matter.” This technical invisibility has slowed mechanistic dissection compared with eukaryotes. Yet, emerging methodologies and tools are making the bacterial condensome increasingly approachable, transforming a longstanding observational challenge into a tractable mechanistic problem.

## THE EMERGENCE OF TOOLS TO PROBE BIOMOLECULAR CONDENSATES IN BACTERIAL CELLS

The intrinsic size and biophysical constraints posed by bacteria make investigating their condensome predominantly an inference challenge: determining whether an observed assembly is a condensate and, if so, characterizing its phase behavior, material state, and regulation in vivo. Addressing these questions requires integrated biochemical, computational, and imaging approaches. While many in vitro assays are readily adapted from eukaryotic systems (reviewed elsewhere; Alberti *et al.*, 2025), computational prediction and live-cell imaging remain uniquely challenging in bacteria because of their small size, distinct protein architectures, and the difficulty of resolving subcellular organization below the diffraction limit.

Computational predictors provide an attractive starting point for condensate discovery, but most were developed using eukaryotic proteins and emphasize scaffold features such as long intrinsically disordered regions (IDRs), low-complexity domains, and prion-like sequences (Saar *et al.*, 2021; Chu *et al.*, 2022; Monti *et al.*, 2025). Although these features contribute to some bacterial condensates, they do not capture the full spectrum of bacterial condensation mechanisms. For example, recent analyses suggest that bacterial and eukaryotic proteomes contain similar fractions of condensate-forming proteins despite major differences in IDR prevalence and architecture, implying that bacteria frequently achieve condensation through mechanisms beyond extensive intrinsic disorder (Ward *et al.*, 2004; van der Lee *et al.*, 2014; Hadarovich *et al.*, 2024). Instead, multivalency often emerges from folded oligomerization domains, short interaction motifs, nucleic-acid binding, or interactions among multiple scaffold and client species (Broedersz *et al.*, 2014; Liu, 2022; Basalla *et al.*, 2023; Trettel *et al.*, 2025b). Consequently, many predictors may perform poorly on bacterial condensates whose assembly relies on mechanisms underrepresented in current training sets. Sparse bacterial representation in most condensate databases further limits the development of mechanism-aware prediction tools (Ning *et al.*, 2020; Wang *et al.*, 2022; Rostam *et al.*, 2023; Rodriguez *et al.*, 2025). Future prediction tools must integrate bacteria-specific sequence, structural, and mechanistic information to identify bacterial condensates beyond canonical eukaryotic paradigms.

Because computational predictions alone cannot establish condensation, candidate assemblies must be validated experimentally. Live-cell imaging provides critical insight into condensate organization and dynamics, but bacterial dimensions impose unique interpretive challenges. Condensates, aggregates, and ordered assemblies can all appear as diffraction-limited foci, obscuring potentially informative differences in morphology and spatial organization. Super-resolution imaging techniques, including Airyscan, photoactivated localization microscopy (PALM), and stochastic optical reconstruction microscopy (STORM) (Tuson and Biteen, 2015), together with single-molecule tracking, help resolve morphology, molecular organization, mobility, confinement, and exchange that cannot be inferred from conventional microscopy alone (Kapanidis *et al.*, 2018). These features may help distinguish among mechanistic models. For example, assemblies may exhibit punctate, filamentous, or surface-associated morphologies that reflect distinct modes of assembly or scaffold organization. Despite the resolution benefits brought by these technologies, the limited repertoire of photoactivatable fluorophores (Lukyanov *et al.*, 2005; Subach *et al.*, 2009; Gaytán and Roldán-Salgado, 2024) precludes simultaneous tracking of multiple components. This limitation is becoming increasingly consequential as the field shifts from identifying condensates to dissecting their multicomponent organization. Moreover, motion blurring, phototoxicity, overexpression, and fluorescent tagging itself (Dörner *et al.*, 2026) can complicate interpretation by altering condensate behavior, particularly for small and transient assemblies. Thus, imaging alone cannot objectively distinguish among material states or establish condensation.

We therefore argue that bacterial condensates should be evaluated using a common set of evidence standards rather than any single experimental criterion (Table 2). These standards probe emergent behaviors arising from the underlying molecular grammar and are therefore most informative when interpreted collectively rather than in isolation. Material state can be assessed by comparison with established aggregation (Govers *et al.*, 2018) and condensation (Hoang *et al.*, 2024) reference systems, together with colocalization of the inclusion-body binding protein IbpA. Condensate dynamics can be assessed by perturbing saturation concentration through changes in temperature or osmolarity, whereas reversibility can be examined by protein dilution following localized laser lysis, inhibition of cell division, or generational dilution (Hoang *et al.*, 2024). Importantly, these approaches require careful interpretation, because they may affect growth, nucleoid organization, and proteostasis.

**TABLE 2:** The criteria for differentiating condensation mechanisms in bacterial systems.

Mechanism	Evidence standard	Methods	Pitfalls	Controls
LLPS	Coexistence of dense and dilute phases	Quantitative fluorescence microscopy; partitioning measurements; phase diagrams	Fluorescent tags alter C_sat_ and condensate properties	Test alternative tags; validate with untagged protein assays
	Concentration buffering (C_sat_)	Measure condensate formation across concentration gradients; quantify dilute-phase concentration above saturation	Assuming increasing condensate size reflects LLPS without demonstrating saturation behavior	Quantitative imaging across concentration, temperature, pH, and ionic strength conditions
	Reversible assembly/disassembly	Protein dilution via localized laser lysis, inhibition of cell division, generational dilution; environmental perturbations (temperature, osmolarity, salt)	Effects caused by altered growth or proteostasis	Monitor cell growth and morphology; compare with known aggregate/condensate controls
	Dynamic molecular exchange	FRAP; single-molecule tracking; fluorescence correlation spectroscopy	Overinterpreting rapid FRAP recovery as proof of liquidity	Use complementary mobility measurements; vary fluorophore/tag placement; compare with known aggregate/condensate controls
	Fusion and relaxation behavior	Time-lapse imaging of fusion events and relaxation kinetics	Fusion alone is not diagnostic because some non-LLPS assemblies can merge	Measure relaxation times and shape recovery; compare with predicted fluid behavior
	Dependence on molecular interactions	Mutational disruption of stickers, oligomerization domains, or interaction motifs; sequence-function analysis	Changes in expression, stability, or localization may indirectly affect condensation	Verify protein abundance, folding, localization, and functional activity
Percolation	Network formation driven by molecular connectivity	Valency mutants; interaction-domain engineering; structural/network imaging	Assuming absence of droplets means absence of organization; confusing dense assemblies with gels	Compare mutants with altered valency but similar expression
	Connectivity-dependent transition near a percolation threshold	Concentration series; quantitative imaging of network formation; phase behavior measurements	Interpreting gradual concentration-dependent changes as percolation without identifying a transition	Determine concentration dependence and threshold behavior; include scaffold variants
	Reduced molecular mobility and emergent elasticity	FRAP; particle tracking; microrheology; optical tweezers (where feasible)	Overinterpreting slow FRAP as evidence of gelation; mobility changes may reflect crowding or aggregation	Combine mobility measurements with mechanical assays; compare with known gels and aggregates
	Elastic network properties	Rheology, microrheology, deformation/recovery assays	Lack of mechanical measurements; relying only on morphology	Directly measure mechanical response and reversibility after perturbation
PSCP	Coexistence of dense/dilute phases together with internal network formation	Quantitative imaging; partitioning measurements; phase diagrams; network analysis	Classifying any dense droplet as PSCP without evidence of internal connectivity	Demonstrate both phase separation and network formation independently
	Concentration buffering (LLPS component)	Measure C*_sat_* and dilute-phase concentration across conditions	Attributing concentration-dependent assembly solely to LLPS	Include concentration series and quantify soluble versus condensed fractions
	Connectivity-dependent material transition	Valency/sticker-spacer perturbations; sequence-function analysis	Confusing aging, aggregation, or maturation with percolation	Test reversible mutations; assess protein solubility and exchangeability
	Viscoelasticity and time-dependent maturation	FRAP, relaxation kinetics, particle tracking, microrheology	Interpreting slow recovery or hardening as aging without distinguishing irreversible aggregation	Compare early and late time points; test reversibility
	Dynamic regulation of material state	Perturb energy flux, ATP/GTP activity, environmental conditions	Ignoring non-equilibrium effects; attributing active regulation to equilibrium phase behavior	Measure energy dependence; include ATPase-deficient or regulatory mutants

Together, advances in computational prediction, quantitative imaging, and perturbation-based validation are shifting bacterial condensate research beyond simply classifying intracellular assemblies as condensates or non-condensates. Instead, these approaches increasingly allow investigators to place assemblies along a continuum of material states while integrating morphology, molecular organization, and dynamic behavior to infer their mechanisms of assembly and biological function.

## ENGINEERING BACTERIAL CONDENSATES FOR BIOTECHNOLOGY

As the field moves from identifying bacterial condensates to understanding the molecular principles governing their behavior, these same principles are beginning to enable rational engineering. The genetic tractability, architectural simplicity, and modularity of bacterial components provide a fertile ground for biomolecular engineering. Underlying these practical benefits is our central contention that condensation is a ubiquitous, yet largely unseen, feature of bacterial biology that remains highly primed for biotechnological exploitation. Synthetic frameworks can exploit condensation to concentrate molecules, organize reactions in space, and couple synthetic functions to cellular inheritance (Stevens and Lasker, 2025). Translating these natural capabilities into predictable engineering outcomes requires a deep understanding of how scaffold architecture and multivalent interactions shape three interconnected parameters: selective partitioning, spatial organization, and material state (Dai *et al.*, 2023b; Galvanetto *et al.*, 2025). Together, this triad governs which molecules enter a synthetic condensate, where it forms within the cell, and how effectively it performs its intended function. The goal is not merely to create condensates, but to program them with properties that harmonize with, or even enhance, the intended functions of the molecules contained within.

Lessons from native bacterial compartmentalization strategies reinforce these principles. BMCs, which assemble through condensate intermediates, can redirect flux through branched metabolic pathways by selectively enriching pathway components (Palmero *et al.*, 2025). Encapsulation peptides and other scaffolds can tune cargo recruitment through interactions whose effective valency and affinity determine loading efficiency and assembly behavior (Oltrogge *et al.*, 2023; Trettel *et al.*, 2025b): overly strong interactions may promote aggregation, whereas weaker interactions may limit encapsulation (Lawrence *et al.*, 2014; Lee and Tullman-Ercek, 2017; Mohajerani *et al.*, 2021). More broadly, these systems highlight the need for synthetic condensates to similarly balance client partitioning, spatial organization, and material state, ultimately mirroring the responsiveness of their natural counterparts.

Selective partitioning is central to many condensate-based applications because function depends on the enrichment of the correct clients, not concentration alone. Engineered RNA-binding scaffolds, such as Pumilio2-elastin-like polypeptide fusions, demonstrate that condensation can either enhance or inhibit translation depending on whether ribosomes are co-recruited with the target transcript (Parker *et al.*, 2022; Shapiro *et al.*, 2025). Productive condensates must therefore enrich intended clients while maintaining access to molecular machinery required for activity. In practice, partitioning can be tuned through scaffold abundance, sticker affinity, oligomerization-driven valency, or client-binding motifs.

Condensation can also be engineered to control spatial organization and inheritance. Natural bacterial systems such as the ParB-*parS*, PopZ, and McdAB demonstrate how distinct condensation mechanisms can be coupled to cellular landmarks and active positioning systems (Babl *et al.*, 2022; Lasker *et al.*, 2022; Basalla *et al.*, 2023). These principles have been repurposed synthetically through platforms such as “MapTag,” which uses the McdAB machinery to actively distribute synthetic cargoes along the nucleoid (Hoang *et al.*, 2025) and PopZ-based condensates that spatially separate reproductive and production-focused cell populations to improve biosynthetic output (Mushnikov *et al.*, 2025). Together, these studies illustrate how condensates can move engineered systems beyond passive diffusion toward programmable intracellular organization.

Material state emerges from the balance between density and network formation described by LLPS, percolation, and PSCP, making it another critical design parameter. Condensates that are too fluid may fail to retain cargo, whereas highly viscous assemblies can impede exchange and cellular fitness. For example, FUS-based condensates used to co-localize enzymes for 2′-fucosyllactose production supported higher output when assemblies remained dynamic rather than viscous or aggregate-like (Ding *et al.*, 2025). Similarly, synthetic coiled-coil triblock systems can be tuned between diffuse, liquid-like, or solid-like states by altering sticker-spacer organization, with more rigid assemblies imposing greater fitness costs (Tomares *et al.*, 2022). Together, these studies demonstrate that material state can be tuned through interaction density, sticker-space architecture, or scaffold valency.

An emerging challenge is that cargos are often treated as passive passengers whose behavior is dictated entirely by the condensate scaffold. This is increasingly consequential as researchers transition into building synthetic condensates with designer functions encoded by enzymatic fusions to scaffolds. Growing evidence suggests that cargo identity—including size, charge, oligomeric state, and interaction potential—can reshape condensate partitioning, dynamics, and material properties (Trettel *et al.*, 2025a; Trettel *et al.*, 2025b; Dörner *et al.*, 2026). Consequently, the same condensation tag may behave differently when fused to different proteins, complicating both interpretation and engineering. This consideration extends even to commonly used fluorescent reporters, which may themselves influence condensate behavior (Dörner *et al.*, 2026). Understanding how cargo properties contribute to condensation therefore represents both a cautionary consideration for experimental design and a potential new design axis beyond scaffold architecture.

Collectively, these studies suggest that bacterial condensate engineering will require simultaneous control over scaffold architecture, client affinity, cargo identity, and material state. Major open questions remain, including how to predict partition coefficients in the crowded bacterial cytoplasm and how to minimize toxicity arising from nonspecific sequestration, altered chromosome organization, proteostasis stress, or metabolic burden. Future synthetic condensates should therefore be evaluated not only by their functional outputs but also by quantitative measurements of partitioning, dynamics, reversibility, and host fitness. Bacterial condensates should be viewed not simply as intracellular containers, but as programmable biomaterials whose physical properties can be engineered to control cellular function.

## A TIMELY OPPORTUNITY: FROM INFERENCE TO ENGINEERING

The central challenge in bacterial condensate research is no longer determining whether condensates exist, but defining the mechanisms that connect scaffold architecture, material state, and biological function. As the field matures, emphasis must shift from cataloging new examples toward establishing evidence standards, identifying recurring molecular grammars, and uncovering the physical principles that govern bacterial condensation.

Addressing these questions will require integrating computational prediction, quantitative imaging, perturbation-based validation, and mechanistic reconstitution. In doing so, we will not only advance our understanding of bacterial cell biology but also establish a foundation for engineering programmable intracellular organization and biomaterials. By uncovering the physical principles that govern this “bacterial dark matter,” we can move from inferring its existence to understanding how bacteria build, regulate, and exploit condensates.

## Abbreviations used:

C_sat_: saturation concentration
C_perc_: percolation concentration
LLPS: liquid-liquid phase separation
PSCP: phase separation coupled to percolation
BMC: bacterial microcompartment
IDR: intrinsically disordered region
PALM: photoactivated localization microscopy
STORM: stochastic optical reconstruction microscopy.
